# Randomized clinical trial to evaluate the effect of a supervised exercise training program on readmissions in patients with myocardial ischemia: a study protocol

**DOI:** 10.1186/1471-2261-13-32

**Published:** 2013-04-25

**Authors:** Núria Santaularia, Josefina Caminal, Anna Arnau, Montserrat Perramon, Jesus Montesinos, Jaume Trapé, Montserrat Abenoza-Guardiola, Pere Guiteras-Val, Tiny Jaarsma

**Affiliations:** 1 Department of Rehabilitation, Althaia Xarxa Assistencial Universitària de Manresa, Manresa, Barcelona, Spain; 2 Department of Medicine, Universitat Autònoma de Barcelona, Barcelona, Spain; 3 Clinical Research Unit, Althaia Xarxa Assistencial Universitària de Manresa, Manresa, Barcelona, Spain; 4 Universitat Internacional de Catalunya (UIC). Sant Cugat del Vallès, Barcelona, Spain; 5 Department of Cardiology, Althaia Xarxa Assistencial Universitària de Manresa, Manresa, Barcelona, Spain; 6 Service of Laboratory Medicine, Althaia Xarxa Assistencial Universitària de Manresa, Manresa, Barcelona, Spain; 7 Department of Social and Welfare Studies, Faculty of Health Sciences, Linköping University, Norrköping, Sweden

**Keywords:** Myocardial ischemia, Exercise therapy, Patient readmission

## Abstract

**Background:**

In recent decades, several studies have assessed the value of cardiac rehabilitation as secondary prevention and have reported substantial reductions in readmissions. However, conclusive evidence is scarce. The present study aims to evaluate the efficacy of a supervised exercise training program for improving percentages of hospital readmission for cardiac causes in patients with myocardial ischemia in the first year after a cardiac event. The effect on all-cause readmission, all-cause mortality, functional capacity, quality of life and adherence to regular exercise is also discussed.

**Methods/Design:**

This study will be conducted as a randomized controlled trial. Eligible patients will be randomly assigned to a control group receiving standard care or to an intervention group which, in addition to standard care, will take part in a supervised exercise training program consisting of three hours a week (spread over three alternate days) of supervised exercise training for 10 weeks. Both groups will perform an exercise stress test and a blood test during the first and third month after hospital discharge. The follow-up period will be 12 months after hospital discharge. The primary outcome measures will be the percentage of patients readmitted, total number of readmissions and length of hospitalization for cardiac disease during the first year after hospital discharge, and time to first hospital admission for cardiac disease.

**Discussion:**

A representative group of hospitalized patients after myocardial ischemia will be studied in order to provide comprehensive data on the potential impact of a supervised exercise training program on hospital readmission rates.

**Trial registration:**

Current Controlled Trials ISRCTN57634424.

## Background

Cardiovascular disease (CVD) is a first-order public health problem all over the world. In 2005, 17.5 million CVD-associated deaths were recorded, and the World Health Statistics 2008 report [1] predicts that this figure will rise to 23.4 million by 2030. In Europe, CVD is the main cause of mortality and has a major impact on health care costs. In Spain, CVD accounts for 35% of all deaths and also has a high morbidity. It is particularly important as a cause of death from age 65 onwards.

Improved diagnosis and more successful treatment of acute illness have increased survival rates after myocardial ischemia (MI). However this also means that the prevalence of CVD is increasing.

In 1964, the World Health Organization (WHO) [2] defined cardiac rehabilitation as "the sum of activity required to ensure cardiac patients the best possible physical, mental, and social conditions so that they may, by their own efforts, regain as normal as possible a place in the community and lead an active life." Since then, several institutions have introduced cardiac rehabilitation in order to offer an integrated physical, psychological and social approach to the treatment of the cardiac patient. In cooperation with the American Association of Cardiovascular and Pulmonary Rehabilitation (AACVPR), the American Heart Association (AHA) published recommendations for cardiac rehabilitation programs (CRP) [3], specifying that these programs should include physical training, aggressive treatment of cardiovascular risk factors, dietary guidance, psychosocial, occupational and physical activity, and the use of all cardioprotective drugs with proven efficacy in secondary prevention. In addition to these recommendations, and where possible, CRPs should include appropriate secondary prevention measures as part of a comprehensive multifactorial approach and should apply the standards of European societies [4]. Secondary prevention through exercise-based cardiac rehabilitation is the approach that has been most successful in reducing morbidity and mortality in coronary artery disease, particularly after MI; CRPs are recommended (Class I), by the European Society of Cardiology, the American Heart Association and the American College of Cardiology [5]. Secondary prevention and cardiac rehabilitation form a single strategy for CRPs designed to reduce heart disease-related morbidity and mortality and the disability it causes by 20-30% [6].

Despite the acknowledged benefits, however, CRPs are currently underused. According to the European Cardiac Rehabilitation Inventory Survey (2008)[7,8], of the 47.5% of the countries that answered the survey, 42.10% (including Belgium, Spain, and Italy) admitted ≤ 30% of eligible patients for phase II of CRP; 15.79% (Luxembourg, Sweden, UK) admitted >30-50% of eligible patients, and 15.79% (Germany, Iceland, Lithuania) admitted >50%. Data were unavailable for the remaining 26%.

Studies on post-MI survival, readmissions and physical rehabilitation date back to 1972 [9]. Most studies have found no significant differences in readmissions post-CRP but in 2005 one study concluded that global community CRP significantly reduced cardiac readmission rates [10]. In 2011, Heran et al. [11] published an updated systematic review of Jolliffe et al’s 2001 study, finding that exercise-based CRPs significantly reduced hospital admissions in the short term (<12 months), and in the same year Lawler [12] published the first meta-analysis showing a statistically significant reduction in MI recurrence with an exercise-based CRP post-MI (OR 0.53; 95% CI: 0.38 - 0.76). Moreover, the few studies of the issue do not focus on identifying the best exercise regimen for reducing readmissions, but on whether the intervention itself affects readmission rates. Only two of the studies presented statistically significant decreases in readmissions [10,12]. The value of readmission as an indicator of effectiveness of exercise lies in the fact that it is an indirect measure of clinical stability, so there is a clear need to study the relation between CRPs and readmission in MI patients.

The aim of this study is to evaluate the efficacy of a supervised exercise training program for reducing cardiac readmissions in patients with MI in the first year after hospital discharge. This study will also provide a detailed evaluation of the program’s effects on total mortality, functional capacity, quality of life and adherence to regular exercise. Our primary hypothesis is that a supervised exercise training program will significantly reduce the incidence of cardiac readmissions in patients with MI in the first year after hospital discharge. The secondary hypothesis is that patients in the intervention group will present significantly better functional capacity, better quality of life, and lower mortality rate.

## Methods/design

### Study design

Open, controlled, randomized, hospital-based clinical trial of patients admitted to the Cardiology Department for MI.

### Study subjects and recruitment

The study will recruit adult patients discharged from hospital with MI as a primary diagnosis. Inpatients will be actively identified by the cardiac nurse and the CRP physiotherapist using daily bed lists.

The inclusion criteria will be: age over 18, diagnosis of MI (myocardial ischemia, pre-infarct angina, cardiac angina, other specific forms of chronic ischemic heart disease or unspecified ischemic heart disease) in the current admission, residence in the catchment area of our hospital, absence of cognitive deficit (Pfeiffer test: 0–2 mistakes), sufficient autonomy to follow the cardiac rehabilitation program (Barthel index >60), and willingness to participate in the study. Signed informed consent will be obtained in all cases. Patients will be excluded if they have symptoms of right heart failure producing pulmonary hypertension or dyspnea caused by severe pulmonary pathology, additional comorbidities affecting the prognosis of cardiac disease, major comorbidities or limitations that could interfere with exercise training program, cognitive impairment or if they do not provide informed consent.

### Randomization

Patients will be randomly assigned to one of two groups: control or intervention. Patients in the intervention group will be included in the supervised exercise training program, while controls will receive standard care. A randomization list will be produced by a computer-generated random-number sequence in blocks of 10 to ensure consistent patient distribution in both groups. The randomization list will be generated by the Clinical Research Unit, which will also control allocation to each group. The researchers will not be aware of the randomization scheme.

Patients will be recruited by the cardiac nurse and the CRP physiotherapist. Neither of them will know the group assignment until the patient has signed the informed consent form. The cardiac nurse or the physiotherapist will then give the name and the patient identification number to the Clinical Research Unit by telephone. After this, participants in both groups will be scheduled for their first visit with the cardiac nurse and for the exercise stress test.

### Study arms

#### Control

Patients assigned to the control group will receive the standard care given at our hospital. During hospitalization, the cardiac nurse or the physiotherapist will assess the cardiovascular risk factors and other clinical variables in each patient. This assessment will check diet, tobacco consumption, hypertension, diabetes, exercise practice, family heart disease, dyspnea, depressive symptoms and activities of daily living. Patients will receive verbal and written information on cardiovascular risk factors from the cardiac nurse or the physiotherapist. This educational information will be related to cardiac disease, cardiovascular risk factors (hypertension, diabetes, cholesterol, triglycerides, tobacco and other drugs, alcohol, stress, overweight, sedentary lifestyle), lifestyle (progressive rehabilitation activities, return to work, driving), diet, physical exercise (phases of readaptation, phases of normal activity, work activity, sexual activity), specific medication in case of cardiac angina (nitroglycerin), pharmacological regimen, complementary assessment (electrocardiogram, echocardiogram, chest X-ray, SPECT, cardiac catheterization, arteriography, electrophysiological study) and treatment of coronary artery disease (pharmacological, coronary angioplasty, surgical treatment). Hospitalized patients will be instructed to do exercises to regain mobility in order to maintain and improve muscular tone and peripheral circulation, and will be taught breathing exercises by the physiotherapist to improve their breathing patterns. Before discharge, the physiotherapist will instruct patients on how to return to physical activity.

#### Intervention

In addition to the usual hospital care, patients randomized to the intervention group will be provided with a supervised outpatient exercise training program, according to the results of the exercise stress test performed one month after hospital discharge and bearing in mind the physical limitations imposed by co-morbid conditions. The program will be performed in the hospital and it will start within the three days after the exercise stress test. It will comprise three hours a week (spread over three alternate days) of supervised exercise training for 10 weeks. The intervention will end after 10 weeks, regardless of whether the patients have completed 30 sessions. Exercise classes will be supervised by a physiotherapist and will consist of 10 minutes of warm-up and muscle stretching, 30 minutes of aerobic exercises (cycloergometer), 15 minutes of isotonic exercises for the upper and lower extremities and 5 minutes of cool-down. Moreover, instructions will be given on self-pulse counting, subjective perception of effort using the Borg scale, relaxation exercises, breathing techniques, postural health and minimizing physical effort. Aerobic exercise intensity will be between 75-90% of the maximum heart rate obtained in the previous exercise stress test and progressing according to the rating of perceived exertion (RPE: Borg scale 11–15). Resistance training will be performed with 10–15 repetitions for three sets, maintaining an RPE of 11–14. The physiotherapist will check that patients are exercising at their prescribed intensity with a pulse oximeter (Quirumed® Health & Care).

After hospital discharge, patients in both groups will have scheduled follow-up visits with a cardiac nurse at the first month, and then after 3, 6 and 12 months (visits 1, 2, 3 and 4 respectively) since hospital discharge in order to control the risk factors, reinforce education for disease control and review adherence to cardiac medication and CRP follow-up.

**Figure 1:**
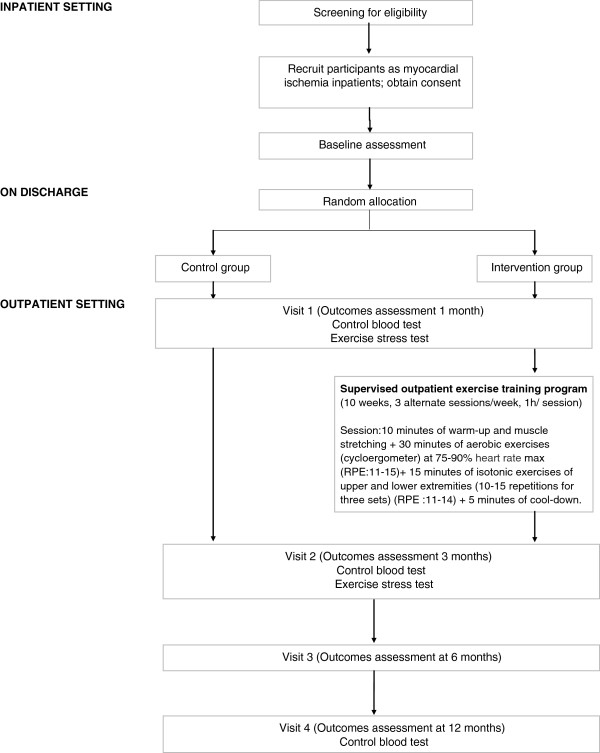
Study design flow-chart.

Control patients will be asked to complete the same outcome measures as the intervention group (See Figure 1).

### Data collection and measures

#### Procedure

Each day, the cardiac nurse and the physiotherapist will check the bed list of patients admitted to the Cardiology Department. They will select the eligible patients after assessing the clinical history and consulting the cardiologist. In the inpatient setting, the cardiac nurse or the physiotherapist will give verbal and written information related to the study to eligible patients and will resolve all the patients’ doubts. These professionals will stress to the patient that the randomization process will decide the group inclusion. Before discharge (visit −1), eligible patients who agree to participate will be asked to give signed informed consent. In this visit, the cardiac nurse will record the following variables: education level, marital status, living situation, employment status and comorbidities (Charlson Comorbidity Index). On discharge (visit 0) the cardiac nurse or the physiotherapist will contact the Clinical Research Unit regarding the randomization process. Once the cardiac nurse or the physiotherapist knows the randomization group, they schedule the patient for the first cardiac nurse visit and for the exercise stress test.

Patients will attend four cardiac nurse visits (visits 1, 2, 3 and 4). During these visits, the cardiac nurse will describe ways of improving cardiovascular risk factors and cardiac medication adherence, and will answer the patients’ questions. The following variables will be recorded: attendance, systolic blood pressure (SBP), diastolic blood pressure (DBP), heart rate, Body Mass Index (BMI), abdominal circumference, Mediterranean diet, depressive symptoms (Yesavage Geriatric Depression Scale), basic activities of daily living (Barthel index), tobacco dependence (Fargeström test), compliance with cardiac medication (Haynes-Sackett test), taking sublingual nitroglycerin, hospital emergency visits, hospital readmissions and mortality. Quality of life (EuroQol5) will be recorded at all visits except visit 3. To address a possible cross-over effect, all patients will be asked to complete a physical activity questionnaire (Capersen and Powell classification) at baseline and in each follow-up visit. This test categorizes physical activity in four levels: sedentary, irregular activity, non-intensive regular activity, and intensive regular activity.

Further, during visit 1, the cardiac nurse will also record the diagnostic reason for referral (MI: location, severity, number of stents, ejection fraction), medication prescribed and level of risk for cardiac rehabilitation. At visit 4, the cardiac nurse will assess whether the patient can return to work. Patients will be administered a satisfaction survey with questions selected from the local public health department’s satisfaction questionnaire.

Blood tests will be performed at visits 1, 2 and 4. The following parameters will be determined: hemoglobin, leucocytes, platelets, urea, creatinine, sodium, potassium, cholesterol, high density lipoprotein cholesterol, low density lipoprotein cholesterol, triglycerides, creatine kinase-MB (CK-MB), C-reactive protein, glycosylated hemoglobin A1c (HbA1c), N-terminal Pro-B-type natriuretic peptide (NTproBNP), microalbumin and calcidiol.

The patient will do the exercise stress test at visit 1 and 2, under the guidance of the cardiologist and the nurse. The Bruce protocol stress test will be used. Before starting, the staff will describe the test to the patient and give some recommendations such as taking a light meal three hours before the test, avoiding stimulating drinks, taking prescribed medication and wearing comfortable shoes and clothes. In this protocol, exercise is performed on a treadmill (QUINTON® Q-STRESS TM 55). The leads of the ECG are placed on the chest wall. The treadmill is started at 2.74 km/hr (1.7 mph) and at a gradient (or incline) of 10%. At three minute intervals the incline of the treadmill increases by 2%, and the speed increases progressively. The exercise stress test will be limited by clinical signs such as arrhythmias and/or ECG changes and clinical symptoms such as general exhaustion, claudication of the legs, chest pain or dyspnea [13].

The following exercise stress variables will be recorded: theoretical maximum heart rate, real maximum heart rate, baseline heart rate, chronotropic index, the level reached on the exercise stress test, and time to recover initial heart rate (1 and 3 minutes). The cardiac nurse will collect blood before and after the exercise stress test in order to determine the level of troponin I.

Additional information will be recorded in the intervention group. Between visits 1 and 2, intervention patients will attend the training sessions. All sessions will be supervised by the physiotherapist. In each session the following values will be compiled: heart rate (initial, maximum, final), oxygen saturation (initial, during session, final), SBP (start, during session, final), DBP (initial, during session, final), degree of dyspnea (initial, final) and the level on the Borg scale. For each patient, the physiotherapist will compile the data from the exercise sessions and the number of physical sessions attended.

The cardiac nurse or physiotherapist will record the cause of withdrawal during the follow-up. The cardiac nurse will collect all data and enter them in a database designed for this study.

Table 1 summarizes the measures and variables according to the timeline.

**Table 1:** Summary of measures and variables according to the timeline

**VISIT**	**−1**	**0**	**1**	**2**	**3**	**4**
**Time (months)**	**−1**	**0**	**1**	**3**	**6**	**12**
**Inclusion/exclusion criteria and informed consent**	X					
**Sociodemographic data** (sex, age, education level, marital status, living situation, employment status) and **comorbidity** (Charlson Index))	X					
**Cardiovascular risk factors** (diet, alcohol, tobacco dependence - Fargeström test - hypertension, diabetes, exercise practice, family heart disease), **dyspnea**, **depressive symptoms** (Yesavage Depression Scale) and **activities of daily living** (Bartel Index)	X		X	X	X	X
**Random allocation**		X				
**Diagnostic reason for referral** (localization, severity, number of stents, ejection fraction)			X			
**Medication prescribed**			X			
**Exercise stress test** (theoretical maximum heart rate, real maximum heart rate, baseline heart rate, chronotropic index, level reached, recovery time of the initial heart rate - 1 and 3 minutes-)			X	X		
**Level of risk for cardiac rehabilitation**			X			
**Control blood test**			X	X		X
**Anthropometric measures** (Body Mass Index, abdominal circumference)			X	X	X	X
**Systolic and diastolic blood pressure** and **baseline heart rate**			X	X	X	X
**Physical activity** (Capersen and Powell classification)			X	X	X	X
**Quality of life** (EuroQol5)			X	X		X
**Cardiac medication adherence** (Haynes-Sackett method) and **taking sublingual nitroglycerin**			X	X	X	X
**Adherence to Cardiac Rehabilitation Program**			X	X	X	X
**Hospital emergencies visits, number of readmissions and total days of hospitalization**			X	X	X	X
**Mortality**			X	X	X	X
**Cause of withdrawal**			X	X	X	X
**Return to work**						X
**Satisfaction with Cardiac Rehabilitation Program** (satisfaction questionnaire of the local public health department)						X

### Assessment of outcomes

#### Primary outcome measures

The primary outcome measures will be the percentage of patients readmitted, total number of readmissions and length of hospitalization for cardiac disease during the first year after hospital discharge and time to first hospital readmission for cardiac disease. The primary outcome will be assessed by an expert committee blind to the patient’s treatment group.

#### Secondary outcome measures

Secondary outcomes are the following: percentage of patients readmitted, total number of readmissions and length of hospitalization for all causes during the first year after hospital discharge, all-cause death, all-cause hospital emergency visits, functional capacity (exercise stress test), quality of life (EuroQol5), adherence to CRP (attendance at follow-up visits), adherence to cardiac pharmacological treatment (Haynes-Sackett test), satisfaction with assistance received (questions selected from the local public health department’s questionnaire, Ambulatory Specialized Care 2008 [14]).

### Demographic and clinical measures

Group allocation, sex, age, education level, marital status, living situation, employment status, main diagnosis, diagnostic reason for referral, location, degree of involvement, number of stents, the ejection fraction, comorbidities (Charlson Comorbidity Index), maximum theoretical heart rate, real maximum heart rate, baseline heart rate, chronotropic index, the level reached on the exercise stress test, time to recover initial heart rate (1 and 3 minutes)), level of risk for cardiac rehabilitation, blood tests, cardiac markers (NTproBNP, troponin I), SBP, DBP, BMI, abdominal circumference, Mediterranean diet, depressive symptoms (Yesavage Depression Scale), basic activities of daily living (Barthel Index), tobacco consumption (Fargeström test), compliance with cardiac medication (Haynes-Sackett method), taking sublingual nitroglycerin, physical activity (Caspersen and Powell classification) and return to work.

### Statistical issues

#### Sample size

To ensure a statistical power of 80% in order to detect differences in the contrast of the null hypothesis (H_0_: equal percentages of cardiac readmissions in the two groups) through a two-tailed chi-square test for two independent samples, with a level of significance of 0.05, and assuming that the percentage of cardiac readmissions in the control group is 25% and the percentage in the intervention group is 12%, each group will require 139 patients. Assuming a 5% loss to follow-up, we estimate that 146 patients will be needed in each arm.

#### Data collection and management

We will collect information on outcome at each stage of recruitment, randomization, treatment allocation, follow-up, and analysis in order to report patient flow according to CONSORT guidelines [15]. We will record the number of patients who meet exclusion criteria, the number of patients who qualified for inclusion but who were not willing to participate, the number of patients assigned to the intervention arm, the number of patients assigned to the control arm, the number of intervention visits, the number of patients who provided follow-up data, the number of patients included in the analysis, and the number of withdrawals.

To ensure that clinical data collection forms are accurately entered into databases, we will periodically validate the information collected in the database (detection of missing and out-of-range values). To maintain the confidentiality of the information, all patients will be identified by study numbers.

#### Statistical analysis

Continuous variables will be summarized using means and standard deviations for normal distributions, and by medians and the 25th and 75th percentiles for non-normal distributions. Categorical variables will be summarized using absolute values and relative frequency. We will test for significant differences among the baseline characteristics of the control and intervention groups. For continuous variables, we will use the Student's *t*-test if both sample groups have normal distribution or the Mann–Whitney *U* test otherwise. We will use the *χ*^2^ test to compare categorical variables (or Fisher’s exact test in 2×2 contingency tables where the expected frequencies are lower than 5).

Percentage of patients readmitted for cardiac disease will be analysed through a two-tailed *χ*^2^ test for two independent samples, and time to first hospital readmission for cardiac disease will be analysed as time to event with Kaplan-Meier estimation and Cox proportional regression models. The deaths occurring without hospitalization will be introduced in the survival analysis as censored data. Event or censored times for all patients will be measured from the time of randomization (visit 0). All information available on the primary and secondary end points will be collected until the time of final contact with the patient, including patients lost to follow-up, at which point follow-up will be censored. Survival data will be presented graphically using the Kaplan-Meier method. We will use the log-rank test to compare the survival curves. To estimate the effect size, we will report the unadjusted hazard ratios (HR) and associated 95% confidence intervals (95% CI) obtained in the bivariate Cox proportional regression models.

To establish whether the allocation group is an independent prognostic factor for cardiac readmission, we will adjust for potential confounders in a multivariate analysis. The model will be adjusted with the significant covariates in bivariate analysis or those that were clinically significant. To estimate the effect size, we will report the adjusted hazard ratios (HR) and associated 95% confidence intervals (95% CI) obtained in the multivariate Cox proportional regression models.

Outcomes will be analysed on an intention-to-treat basis. A two-sided α-level of 0.05 will be considered statistically significant. Data will be analysed using IBM SPSS Statistics for Windows v.20 and Stata v.10.

### Ethical approval

The protocol has been approved by the independent Ethical Committee of Clinical Research of our hospital. Before recruitment, all patients will provide written, informed consent to participate. The study will comply with all local legal and regulatory requirements and with the Declaration of Helsinki. Trial registration on Current Controlled Trials is ISRCTN57634424 a date 30/04/2010.

## Limitations

One limitation of this study is its open design, since it will not be possible to blind the intervention and both patients and staff will know to which group they have been assigned. This may affect the degree of response, especially in the control group. Another limitation may be the difficulty of including patients due to the large size of the catchment area and its mainly rural nature. This may restrict the inclusion process due to the distance and transport problems, and may limit the external validity.

## Discussion

The objectives pursued by this project have a direct impact on the welfare of patients with myocardial ischemia. Reducing cardiac readmissions and improving quality of life and functional status of the patient accelerate return to normal life and shorten convalescence time. In addition, during admission and follow-up, the patient and family will receive detailed information on lifestyle habits, physical exercise and aspects to consider in order to favour active control of the disease by the patient and to increase safety, both highly important factors for reducing the number of readmissions.

From the standpoint of health planning and management, the findings of the study may entail substantial savings in the budgets of health services in the country, reducing readmissions and the total length of hospitalization in a disease as frequent as myocardial ischemia. Thus, this clinical trial may encourage the systematic implementation of supervised exercise training program on cardiac rehabilitation programs in our environment. The intervention will not be overly complex and can be performed easily by well-trained health professionals, and could be widely applied if it demonstrates a clinically relevant degree of efficacy. In the reference area of our hospital, there is no central unit that organizes secondary prevention and supervised exercise training programs for cardiac patients. As a result, our patients are not currently undergoing treatment at other centers.

This multidisciplinary experimental clinical study will promote research in groups of MI patients who are representative of usual clinical practice. It includes validated health-related quality of life outcome measures and hospital admission.

## Abbreviations

AACVPR: American Association of Cardiovascular and Pulmonary Rehabilitation; AHA: American Heart Association; BMI: Body Mass Index; CK-MB: Creatine Kinase-MB; CRP: Cardiac Rehabilitation Program; CVD: Cardiovascular Disease; DBP: Diastolic Blood Pressure; HbA1c: Glycosylated Hemoglobin A1c; ISRCTN: International Standard Randomized Controlled Trial Number; MI: Myocardial ischemia; NTproBNP: N-terminal Pro-B-type Natriuretic Peptide; post-MI: Post-Myocardial Ischemia; RPE: Rating of perceived exertion; SBP: Systolic blood pressure; SPSS: Statistical Package for the Social Sciences; WHO: World Health Organization.

## Competing interests

The authors declare that they have no competing interests.

## Authors’ contributions

NS contributed to the conception of the idea for the study. All the authors developed the study protocol, the organization and sought funding and ethical approving. NS, JC, AA, JM and TJ drafted the manuscript. All the authors have read the draft critically, have made contributions, and have approved the final text.

## Pre-publication history

The pre-publication history for this paper can be accessed here:

http://www.biomedcentral.com/1471-2261/13/32/prepub
